# Super-resolution fluorescence imaging of intracellular mutant huntingtin protein reveals a population of fibrillar aggregates co-existing with compact perinuclear inclusion bodies

**DOI:** 10.1186/1750-1326-8-S1-O18

**Published:** 2013-09-13

**Authors:** Steffen J Sahl, Lucien E Weiss, Lana Lau, Judith Frydman, WE Moerner

**Affiliations:** 1 Department of Chemistry, Stanford University, Stanford, CA, USA; 2 Department of Biology Stanford University, Stanford, CA, USA

## Background

The identities of toxic aggregate species in Huntington’s disease (HD) pathogenesis remain unclear. While polyQ-expanded mutant huntingtin (Htt) is known to accumulate in compact inclusion bodies inside neurons, this is widely thought to be a protective coping response that sequesters misfolded conformations or aggregated states of the mutated protein.

## Materials and methods, results

To define the spatial distributions of fluorescently-labeled Htt-exon1 species in the cell model PC12m (terminally differentiated into sympathetic-neuron-like cells with nerve growth factor), we employed highly sensitive single-molecule-based and stimulated emission depletion (STED) super-resolution fluorescence imaging modalities. In addition to inclusion bodies and the diffuse pool of monomers and oligomers, fibrillar aggregates ~100 nm in diameter and up to ~1-2 µm in length were observed for pathogenic polyQ tracts (expression experiments with 46 and 97 repeats) after targeted photo-bleaching of the inclusion bodies [1]. These short structures bear a striking resemblance to fibers described *in vitro *[2]. We identified a sharp cut-off behavior of maximum fibril length and documented the ensuing bundling of these fibers into denser arrangements of varying complexity, both in the cytosolic space and inside the neuritic processes.

## Conclusions

Definition of the diverse Htt structures in cells will provide an avenue to link the impact of pharmacological agents to aggregate populations and morphologies. The latest observations w.r.t. co-localization of Htt with various quality control proteins such as the chaperone Hsp70 will be presented.
